# The efficacy of medical management of leiomyoma-associated heavy menstrual bleeding: a mini review

**DOI:** 10.1016/j.xfre.2023.10.003

**Published:** 2023-10-13

**Authors:** Mariam Barseghyan, Jennifer Chae-Kim, William H. Catherino

**Affiliations:** a Obstetrics and Gynecology, MedStar Washington Hospital Center, Georgetown University, Washington, District of Columbia; b Eunice Kennedy Shriver, National Institute of Child Health and Human Development, National Institute of Health, Bethesda, Maryland; c Gynecologic Surgery and Obstetrics, Uniformed Services University of Health Sciences, Bethesda, Maryland

**Keywords:** Heavy menstrual bleeding, leiomyoma, medical management, uterine fibroids

## Abstract

Leiomyomas, or fibroids, are benign uterine tumors that are commonly associated with abnormal uterine bleeding-L particularly heavy menstrual bleeding (HMB). Treatment options include expectant, medical, image-guided, and surgical. Medical management of HMB is the preferred first-line treatment and includes nonsteroidal anti-inflammatory drugs, contraceptive hormones, tranexamic acid, levonorgestrel intrauterine system, gonadotropin-releasing hormone (GnRH) antagonists and antagonists, selective progesterone receptor modulators, selective estrogen receptor modulators, and aromatase inhibitors. Although alternatives such as vitamins and supplements have been suggested, there is currently a lack of robust evidence of their efficacy. Many of these therapies treat the symptoms rather than the underlying pathology. Progestin-based therapies are the most commonly utilized, although research supporting their effectiveness in the treatment of HMB is modest. Although GnRH agonists and antagonists, which are federal drug administration-approved therapies, provide substantial improvement in abnormal uterine bleeding-L with HMB, the effects typically last for the duration of therapy. Patients may also face financial barriers to GnRH analog therapy. Future studies are required to delineate the nonhormonal treatment options and the long-term management of leiomyoma-associated HMB.

## Introduction

Leiomyomas, or fibroids, are benign uterine tumors comprised leiomyocytes presumed to derive from smooth muscle cells. Leiomyomas vary in size, location, and symptomatic manifestation. Most leiomyomas are asymptomatic and found incidentally. Although up to 70% of women will be diagnosed with leiomyomata by the end of their reproductive lifespan, it is estimated that only 25% of them require intervention (1). Leiomyomas are often found in association with abnormal uterine bleeding (AUB), which is defined as uterine bleeding during reproductive years in the absence of pregnancy that is abnormal in regularity, volume, frequency, or duration (2). Heavy menstrual bleeding (HMB) is the most common presenting symptom of women with fibroids, whereas bulk symptoms such as pelvic pressure, constipation, and urinary frequency are less common and are associated with larger leiomyoma size (1). Fibroids are often palpated on a careful bimanual examination, although palpated fibroids may not always be symptomatic. For more precise delineation of the location and size of fibroids, as well as evaluation of submucosal fibroids, which cannot be palpated on the examination, transvaginal ultrasonography is utilized. Sonohysterography, hysteroscopy, and magnetic resonance imaging could be further used to classify leiomyomas according to the International Federation of Gynecology and Obstetrics subclassification system to describe the precise location of leiomyomas. International Federation of Gynecology and Obstetrics denotes HMB associated with submucosal fibroids as AUB-Lsm and AUB-Lo for other types of fibroids (1, 2). Myometrial conditions, such as the presence of leiomyomas, lead to the changes in endometrial function resulting in AUB (3).

Treatment of leiomyomas is multifaceted; options include expectant, medical, image-guided, and surgical management. Expectant management entails symptomatic monitoring with regular follow-ups as clinically indicated and is typically reserved for women with no or minimal symptomatic burden. Surgical management is primarily aimed at treating HMB, bulk symptoms, or to improve fertility outcomes. Hysterectomy is the definitive management of both bulk symptoms and HMB but is not fertility-sparing. Uterine artery embolization and endometrial ablation are commonly utilized procedural methods of treatment for symptomatic leiomyomas; however, endometrial ablation is contraindicated if future fertility is desired, whereas the effect of uterine artery embolization on fertility is unclear. Myomectomy is a fertility-sparing surgical treatment available, although there are risks of adhesion formation and implications for the route and timing of future pregnancy delivery. Laparoscopic and transcervical radiofrequency ablation as well as high-intensity focused ultrasound are emerging technologies for the treatment of symptomatic fibroids; however, their therapeutic effects may be limited (1).

Medical management is the preferred first-line treatment for AUB-L for many patients. Medical treatment options that have been assessed scientifically include nonsteroidal anti-inflammatory drugs (NSAIDs), tranexamic acid (TXA), systemic contraceptive steroid hormones, levonorgestrel intrauterine system (LNG-IUS), gonadotropin-releasing hormone (GnRH) antagonists, GnRH agonists, selective progesterone receptor modulators (SPRMs), selective estrogen receptor modulators, and aromatase inhibitors. Complementary therapies include vitamins and supplements which require further investigation.

No single medical treatment is considered superior to other options in the treatment of AUB-L; however, certain medical therapies have more evidence of benefit. The effects of most agents are targeted to alleviate HMB patterns, rather than treating fibroid burden directly. Some forms of medical treatment can affect fibroid size, but there is no available treatment option that will permanently cease fibroid tumor growth. In this mini review, we present evidence for various medical management options for leiomyoma-associated HMB while highlighting important considerations for each treatment option.

## Nonsteroidal anti-inflammatory drugs

Nonsteroidal anti-inflammatory drugs inhibit cyclooxygenase, thus preventing the transformation of arachidonic acid to prostaglandins involved in HMB. Like antifibrinolytic medications, this group of medications offers nonhormonal treatment for HMB and should be considered particularly by women who wish to conceive. A Cochrane review highlighted that NSAIDs reduce HMB compared with placebo but are less effective than TXA (discussed later) (4). However, most studies investigating the efficacy of NSAIDs are in patients with no pathological causes of HMB, which makes extrapolation of its efficacy in fibroid-induced HMB unclear.

## Tranexamic acid

Tranexamic acid is a synthetic derivative of the amino acid lysine. It reversibly blocks lysine-binding sites on the plasminogen molecule therefore exhibiting an antifibrinolytic effect. It is available in intravenous and oral formulations. It is typically used to control acute bleeding but may also be used for outpatient management of HMB in patients who are unable to take hormonal treatment. A pivotal randomized control trial investigating the effects of TXA on HMB demonstrated that TXA significantly reduced AUB-L-associated HMB; however, the study did not characterize the leiomyomas (5). Tranexamic acid does not treat the fibroid directly, nor are there long-term treatment data.

## Systemic contraceptive steroid hormones

In addition to pregnancy prevention, combined oral contraceptive (COC) pills have other functional effects. They control HMB by preventing ovulation and inhibiting endometrial proliferation because of their progestin-dominant formulation, thus leading to the formation of a thinner endometrial lining and less menstrual effluent.

There are limited direct data to support the effectiveness of contraceptive hormone use to manage AUB-L induced HMB as most studies using COCs investigate women with AUB-O (ovulatory) and AUB-E (endometrium). Moreover, there is no evidence that the use of contraceptive steroid hormones improves the bulk symptoms associated with uterine leiomyomas. Studies with cell culture and animal models have historically demonstrated that estradiol is one of the factors for leiomyoma growth. In addition, studies demonstrate that mitotic activity in leiomyomas is higher during the secretory phase of the menstrual cycle predominated by progesterone. The growth of a leiomyoma correlates with the progesterone receptor content and the proportion of smooth muscle in a leiomyoma. Uterine leiomyoma xenograft studies have also demonstrated that volume maintenance and growth of human leiomyomas are progesterone dependent (6).

The impact of fibroid risk attributed to the past or current hormonal contraceptive use has been evaluated in different observational studies with inconsistent results. Although there are no robust trials demonstrating the efficacy of COCs for the treatment of AUB-L-associated HMB, a trial of these medications may be useful for some women. In a recent study, women with AUB-L with fibroids less than 5 cm in size were randomized into two groups, those who received combined hormonal vaginal rings and those who received ultra-low-dose COCs for the management of HMB. Reduction in menstrual blood loss was 72% and 62% at 6 months while on treatment and 71% and 55% 3 months after stopping the treatment between the vaginal ring vs. COC groups, respectively, suggesting short-term benefit of the vaginal ring and COC treatment (7). These data also imply that a quarter to one third of patients do not benefit from treatment, and those that initially respond have decreasing benefit over time.

A recent review evaluating progestogen treatment for uterine fibroids concluded that there is a lack of evidence to demonstrate the efficacy of progestogens in the treatment of fibroid-associated HMB (8). A Cochrane systematic review found that a 21-day regimen of norethisterone (also known as norethindrone) results in a significant reduction of ovulatory HMB, but not if it is administered during the luteal phase (9). Furthermore, its efficacy in fibroid-associated HMB is unclear and it is typically prescribed for short-term use because of the high incidence of side effects.

## Levonogestrel intrauterine system

The 52-mg LNG-IUS is one of the most effective forms of contraception that has also shown efficacy in the treatment of AUB. Levonorgestrel intrauterine system reduces menstrual bleeding by inducing endometrial decidualization and atrophy and has been found to decrease menstrual bleeding in patients with and without leiomyomas. However, there is insufficient evidence to support the use of LNG-IUS for the treatment of leiomyoma-induced bulk symptoms (10).

A single-center randomized clinical trial evaluated 58 women with AUB-L-associated HMB who received LNG-IUS or COC concluded that LNG-IUS was more effective in reducing menstrual bleeding than COC (11). Most studies evaluating the use of LNG-IUS for the treatment of fibroid-related HMB demonstrated its effectiveness in reducing menstrual blood flow. However, these studies often excluded patients with submucous fibroids that caused uterine cavity distortion, which is a relative contraindication for LNG-IUS insertion. A retrospective cohort study that evaluated 41,561 women aged 18–54 with fibroid-related HMB over 13 years concluded that women were more likely to persist with their initial therapy of a long-acting reversible contraceptive such as LNG-IUS compared with other medical options including COCs, although most of patients’ first-line therapy was COCs (12). The study showed that 21.6% of women discontinued the LNG-IUS therapy, which could last for 3–5 years, leading to a return of symptoms. There appear to be ample studies comparing COCs to LNG-IUS with most demonstrating significantly decreased menstrual bleeding and improved quality of life with the use of LNG-IUS, but data are sparse on the efficacy in the setting of AUB-L-related HMB.

## GnRH analogs

Other therapies for the medical management of leiomyoma include GnRH agonists or antagonists. These medications act on the hypothalamic–pituitary–ovarian (HPO) axis to induce a hypoestrogenic state. Gonadotropin-releasing hormone agonists stimulate the GnRH receptor, resulting in an initial “flare” effect of gonadotropins; however, continuous stimulation results in receptor desensitization and ultimately downregulation of the HPO axis. Gonadotropin-releasing hormone antagonists block the GnRH receptor, resulting in downregulation of the HPO axis and immediate suppression of gonadotropin production. Given their side effect profile, GnRH agonists or antagonists were initially recognized as second-line therapies, and the only injectable analog, leuprolide acetate, was initially federal drug administration (FDA) approved to be used temporarily in the perioperative period to decrease blood loss before myomectomy or hysterectomy. Hormonal add-back therapy with estrogen and progestins can be used alongside GnRH agonist or antagonist therapy to lessen the hypoestrogenic effects of these medications. Leuprolide acetate was FDA approved for up to 6–12 months’ use, without or with add-back therapy, for treatment of AUB-L (13). Newer oral antagonists, elagolix and relugolix, are specifically FDA approved for the treatment of AUB-L with add-back therapy (estradiol 1 mg and norethindrone acetate 0.5 mg) for up to 24 months. Several studies have shown significantly decreased amount of menstrual bleeding, pelvic pain, and leiomyoma size in women with AUB-L who were treated with elagolix or relugolix, compared with placebo (14, 15).

## Selective progesterone receptor modulators

Mifepristone, also known as RU486, is the original progesterone receptor modulator, which demonstrated improvement of leiomyomata-associated quality of life and reduction in fibroid size (16). Other progesterone receptor modulators such as ulipristal (UPA) acetate have been shown to be efficacious in short-term treatment of AUB-L in a few studies outside of the United States. However, reports of hepatotoxicity with UPA administration resulted in its temporary suspension in the United Kingdom where it is currently used for intermittent treatment of moderate to severe symptoms of fibroids (17). In the United States, UPA is only FDA approved for use as emergency contraception (18). Vilaprisan, asoprisnil, and telapristone are SPRMs that were used in clinical trials only and demonstrated a decrease in the fibroid size and related HMB. However, all further trials with SPRMs have been halted because of safety concerns (19).

## Selective estrogen receptor modulators

Selective estrogen receptor modulators are nonsteroidal agents that bind to the estrogen receptor and exhibit either agonist or antagonist effects depending on the tissue. The most well-studied agents in this class are tamoxifen and raloxifene. Tamoxifen is primarily used in the treatment of breast cancer. It works as a partial agonist on the endometrium and provides an unopposed estrogen stimulus to the endometrium, increasing the risk of atypia and cancer. Given the expression of estrogen receptor on leiomyomatous tissue, significant fibroid growth was observed in women with breast cancer receiving tamoxifen treatment (20). Raloxifene, which exhibits estrogen-antagonistic action in the uterus and breast tissues and is used as an antiresorptive agent in patients with osteoporosis, demonstrated significant fibroid volume reduction in postmenopausal women, but not premenopausal women (21). Ormeloxifene, originally developed in India as an oral contraceptive administered twice weekly, has been shown to be effective in treating AUB-L in 72% of patients after 6 months of the treatment compared with 8% in women with COCs. This study also showed significant leiomyoma volume increase in both groups (22) Currently, selective estrogen receptor modulators have insufficient data to be used clinically for the treatment of AUB-L.

## Aromatase inhibitors

Aromatase converts androgens into estrogens and because there is an intrinsic capacity of leiomyomas to produce estrogens because of the expression of aromatase, the inhibitors of this enzyme have been assessed therapeutically. A Cochrane systematic review identified only one randomized control trial that demonstrated a nonsignificant difference between letrozole and GnRH agonists on fibroid volume reduction at 12 weeks of treatment. The review concluded that the evidence to support the use of aromatase inhibitors in the treatment of women with uterine fibroids is insufficient (23). As such, these compounds should be used only in clinical trials.

## Vitamins and supplements

It has been proposed that Vitamin D could inhibit fibroid growth as a potential antitumor compound. In a recent randomized controlled trial, no statistically significant decrease in the fibroid volume was observed in a group that received Vitamin D for 12 weeks compared with the control group in which a significant increase in the fibroid volume was observed implying that Vitamin D consumption may inhibit fibroid growth (24). Similarly, the green tea extract, epigallocatechin gallate, has been shown to inhibit and potentially lead to the apoptosis of fibroid cells. A randomized pilot-controlled clinical study demonstrated that patients who received epigallocatechin gallate had a significant reduction in fibroid volume and associated HMB compared with the placebo group (25). Such studies are limited, and no recommendations can be made without further investigation.

## Conclusion

Leiomyomas are one of the most common reasons for HMB. Although there are many treatment options for the management of fibroid-associated HMB, there is no single universal treatment option. Medical management is the first line and the most preferred mode of treatment for most patients, and although options are improving for patients, there is no one therapy that is ideal for all. These options and various considerations are presented in Table 1. The clinically efficacious FDA-approved oral therapies, elagolix and relugolix, directly treat the leiomyoma and provide substantial improvement in AUB-L with HMB. However, from a cost and accessibility perspective, progestin-releasing options will likely remain first line. The available medical treatment options need to be individualized focusing on therapies with clinical evidence of benefit for women suffering from AUB-L with HMB, rather than HMB from other causes. Furthermore, additional research is required for the assessment of optimal therapies for long-term treatment, as fibroid symptomatology will return after cessation of effective therapies.

**Table 1:** Medical treatment of heavy menstrual bleeding (HMB) because of abnormal uterine bleeding secondary to leiomyomas (AUB-L).

Medication class	Mechanism of action	Effect on AUB-L-induced HMB	Take home points
Nonsteroidal treatment			
NSAIDs	Inhibit cyclooxygenase lowering prostaglandin levels	Reduce HMB compared with placebo but uncertain evidence for AUB-L HMB	• Often first-line therapy • An option for women considering initial nonhormonal treatment
TXA	Reversibly blocks lysine-binding sites on the plasminogen molecule exhibiting an antifibrinolytic effect	Significantly reduces menstrual blood loss in women with uterine fibroids compared with placebo	• Used to control bleeding in acute as well as chronic settings
Vitamins and supplements	Various	Vitamin D and green tea extract potentially lead to decrease in HMB, insufficient evidence	• Use should be limited to clinical trials
Gonadal steroidal treatment			
Systemic contraceptive steroid Hormones	Prevent ovulation, inhibit endometrial proliferation	No robust trials demonstrating the efficacy	• A trial of these medications may be useful for some women
LNG-IUS	Induces endometrial decidualization and atrophy	Data are sparse on the efficacy in the setting of AUB-L related HMB	• May be superior to contraceptive steroid hormones
Nonsteroidal compounds affecting innate hormonal pathways			
GnRH analogs	Act on the hypothalamic–pituitary–ovarian axis to induce a hypoestrogenic state	Directly treat the leiomyoma and provide substantial improvement in AUB-L with HMB	• FDA approved treatment options for AUB-L include leuprolide acetate, elagolix, and relugolix • Elagolix and relugolix with add-back therapy up to 24 mo use • Directly treat disease
SPRMs	Bind to the progesterone receptor and exhibit either agonist or antagonist effects depending on the tissue	Outside of the United States, ulipristal has been shown to be efficacious in short-term treatment of AUB-L	• Not available for leiomyoma treatment in the United States
SERMs	Bind to the estrogen receptor and exhibit either agonist or antagonist effects depending on the tissue	Insufficient data to be used clinically for the treatment of AUB-L	• Use should be limited to clinical trials
AI	Inhibit conversion of androgens into estrogens	Insufficient evidence to support use for AUB-L	• Use should be limited to clinical trials

AI = aromatase inhibitor; GnRH = gonadotropin-releasing hormone; LNG-IUS = levonorgestrel intrauterine system; NSAID = nonsteroidal anti-inflammatory drug; SERM = selective estrogen receptor modulator; SPERM = selective progesterone receptor modulator; TXA = tranexamic acid.

## Declaration of Interests

M.B. has nothing to disclose. J.C.-K. has nothing to disclose. W.H. C. reports consulting fees from Bayer and Myovant; travel support from Myovant; Editor-in-Chief, F&S Science; leadership role American Board of Obstetrics and Gynecology outside the submitted work.
